# Creativity and (global, ethnic, host) cultural identifications: An examination in migrant and host national samples

**DOI:** 10.3389/fpsyg.2022.1007034

**Published:** 2022-11-04

**Authors:** Elia Soler Pastor, Magdalena Bobowik, Verónica Benet Martínez

**Affiliations:** ^1^Department of Political and Social Sciences, Pompeu Fabra University, Barcelona, Spain; ^2^Department of Interdisciplinary Social Sciences, Utrecht University, Utrecht, Netherlands; ^3^ICREA (Catalan Institution for Research and Advanced Studies), Barcelona, Spain

**Keywords:** global culture identification, host culture identification, ethnic culture identification, creativity, immigrant sample, migration

## Abstract

We live in an era of unprecedented interconnectivity and challenges (e.g., climate change, pandemics) that require global mindsets and creative approaches. While research on global identification has increased in recent years, the question of whether it can facilitate creativity remains largely unexplored. Moreover, despite the evidence linking multicultural experiences and global identities, migrant populations have been overly underrepresented in this area of research. We examine the association between global culture identification and creativity in the Alternate Uses Test, across two different samples residing in Spain: a host national and majorly student sample (*N* = 326) and a culturally diverse immigrant sample (*N* = 122). Additionally, we test the predictive value of ethnic identification (in both samples) and host culture identification (in the immigrant sample). Regression analyses reveal that global culture identification positively predicts creativity among host national participants, and host culture identification predicts creativity among immigrant participants. Our results suggest that developing a cultural identity that transcends the one acquired through enculturation (i.e., global culture identification for the host national sample, host culture identification for the immigrant sample) has the potential of facilitating creative behavior.

## Introduction

Nowadays, people may feel connected with others who live in any part of the world, far beyond their own current geographical location and related local communities and personal relationships (McFarland et al., 2019). Scholars have defined and measured such feelings of global identification using many related constructs and instruments, including identification with a global or world community (Buchan et al., 2011; Marcus et al., 2017), being a world citizen (Smith et al., 2017), intercultural identity (Sussman, 2000), identification with all humanity (IWAH, McFarland et al., 2012), cosmopolitan orientation (Leung et al., 2015), and global-human identity (Türken, 2006; Nickerson and Louis, 2008). However, in parallel to feeling identified with people all around the world, people may also feel part of a global culture, including events, practices, models of lifestyle and consumption, and information (symbols, images) that are developed in different parts of the world and shared transnationally (see Arnett, 2002; Bobowik et al., 2022). Multicultural experiences, that underlie the development of such superordinate identifications, are known to promote creative thinking and behavior (Cheng and Leung, 2013; Fee et al., 2013; Crisp and Gocłowska, 2014; Cheng and Tan, 2017; Chua, 2018). Scholars have found that the more personally relevant the multicultural experience is, or the stronger the identification with the different cultures involved, the greater the creative benefits it will produce (Tadmor et al., 2012a; Cheng and Tan, 2017; Chua, 2018).

Nevertheless, little attention has been paid to the link between a superordinate identification with a global culture and individual creative behavior. Moreover, research has rarely focused on global identification among underrepresented sectors of the society such as migrants. To address these gaps, we hereby examine the role of identification with a global culture in predicting creative behavior across two samples: host nationals and immigrants. Furthermore, we study its relationship with creativity comparatively, in tandem with identification with the host culture (for immigrants) and identification with one’s ethnic culture of origin.

### Levels of identification resulting from acculturation

From a social constructivist perspective, identities are constructed in relation to and in interaction with other people and their systems of representation (Gläveanu and Tanggaard, 2014), and derive from one’s membership or attachment to a social group (Hamer et al., 2021). In line with this approach, all individuals go through the process of *enculturation* when they are in contact with and internalize their origin culture (e.g., its salient values, norms, and rituals) through early socialization (Kim and Alamilla, 2007). This way, they become competent members of their own cultural group and acquire a sense of identity attached to their ethnic/heritage culture (Ferguson et al., 2016).

In parallel, in multicultural societies, people go through *acculturation* when they are in contact with a different culture (see Sam and Berry, 2010). Acculturation is usually studied in the context of migration, but it can more broadly be understood as the psychological processes that people go through when adapting to a new cultural group membership. As a result, individuals may develop dual or multiple cultural identities (Crisp and Gocłowska, 2014). For example, immigrants may develop identification to the host culture while preserving their ethnic identity.

Individuals who develop—through acculturation—dual (or multiple) identities are more likely to integrate these different cultural representations into wider and higher-order identity configurations, such as European or global human identities (Amiot et al., 2007; Crisp and Gocłowska, 2014). Among immigrants and expatriates, global identity constitutes an identity management strategy linked to the integration of both home and host cultures (i.e., bicultural identity integration, Benet-Martínez and Haritatos, 2005; Bobowik et al., 2022) or to a “balanced identity” pattern (Kohonen, 2008). There is also evidence (with people who study abroad) that the longer the engagement with a second culture, and the more significant the cultural differences between host and home cultures, the greater the increase in global or world mindedness (Douglas and Jones-rikkers, 2001; see also Crisp and Gocłowska, 2014). Thus, acculturation processes derived from globalization or contact with multiple cultural contents could also result in identification with a global community or with all humanity (Arnett, 2002; Chen et al., 2008; Reysen, 2022), which reflect the more inclusive and superordinate levels of self-categorization (McFarland et al., 2012, 2013, 2019).^1^

Experiences of cultural diversity encourage the development of these more inclusive, plural, and transnational identity configurations (Lubbers et al., 2007; Sparkman and Hamer, 2020; Reysen, 2022). Consequently, the development of global mindsets has often been studied in multicultural contexts such as expatriate assignments (Fee et al., 2013), participation in multicultural teams (Erez et al., 2013) and global networks (Grimalda et al., 2018), exposure to and contact with different cultural elements or people (Sparkman and Eidelman, 2018; Sparkman and Hamer, 2020), and cultural diversity in one’s personal social network (Lubbers et al., 2007; Mao and Shen, 2015; Bobowik et al., 2022).

### Creativity as a potential outcome of identification processes

Creativity is the most widely studied intrapersonal outcome of multicultural experiences (e.g., Leung et al., 2008; Leung and Chiu, 2010; Crisp and Gocłowska, 2014) and multicultural identities (Cheng et al., 2008; Kharkhurin, 2009; Maddux et al., 2009; Saad et al., 2012; Tadmor et al., 2012a). These types of experiences stimulate creative thought because they require individuals to adopt new ways of thinking that often collide with their previous mental schemas (Crisp and Gocłowska, 2014), pushing them to contrast and connect culturally distant ideas. This results in recategorization processes that ultimately enhance their cognitive flexibility (Ritter et al., 2012; Gocłowska et al., 2018; Chirico et al., 2020), integrative complexity (Cheng et al., 2011), and creative skills (Kharkhurin, 2011).

The stronger the identification with the different cultures, the greater potential for creativity it produces, because individuals will be more motivated to cognitively process and resolve the cultural discrepancies perceived (Cheng et al., 2011; Tadmor et al., 2012b; Cheng and Tan, 2017; Chua, 2018). In this line, bicultural individuals show more complex cultural representations than monoculturals (Benet-Martínez et al., 2006), and those who perceive their different cultural identities as compatible and in harmony (vs. incompatible and in conflict) better integrate ideas from their different cultures in creativity tasks (Cheng et al., 2008; Chua, 2013; Cheng and Tan, 2017).^2^

Similar to the effect of multicultural experiences “loosening” categorical thinking and allowing for new and more flexible representations, global citizenship values are thought to foster greater acceptance to diverse ideas and ways of perceiving the world (Tidikis and Dunbar, 2019).^3^ Consistent with this idea, evidence shows that global identification is linked to outcomes that involve greater openness to others and acceptance of new ways of perceiving world, such as concern for human rights and global issues (Buchan et al., 2011; McFarland et al., 2012), cooperative behavior (Buchan et al., 2011; Grimalda et al., 2018), and volunteering (McFarland, 2016). Acquiring a new cultural framework, thus, stimulates the flexible and complex type of thinking processes that are the foundation for creativity, and individuals who can integrate newly developed cultural identities with their ethnic or origin culture are more likely to reap the creative benefits of multiculturalism (Cheng et al., 2011). The development of wider (e.g., global) identities may as well promote creativity by broadening the base of ideas, interests, norms, values, and behaviors that are accepted, incorporated and cognitively accessible (Crisp and Gocłowska, 2014).

In comparison to more inclusive levels of identifications, we know little regarding ethnic identification and creative behavior. Given that novelty is an important component of creativity, strong attachment to one’s origin ethnic culture may hinder creative behavior, since it may reinforce and rigidize old and pre-established cultural representations (see Kharkhurin, 2011). Yet, some authors suggest that identification with a national/local/ethnic culture can also derive in creativity (Sun, 2018; Mehta, 2019),^4^ especially when conceptualized as national attachment or *constructive patriotism* (i.e., love for one’s country) vs. *glorification* or superiority of one’s home nation over other nations (see Clerkin, 2013; Tidikis et al., 2018). Only a few recent studies examine the associations between global or local (ethnic) identifications and creativity, and results are inconclusive. Tidikis and Dunbar (2019) found that global citizenship predicted creativity in different domains (i.e., everyday, scholarly and mechanic/scientific creativity). In contrast, Tidikis et al. (2018) showed that a global prime condition increased creativity only among US participants, while national ethnic identification increased creativity among Lithuanian participants. In any case, with the exception of biculturalism, cultural identity processes have rarely been considered to shape creative behavior directly (Gläveanu and Tanggaard, 2014), and, to our knowledge, the role of global vs. ethnic culture identification, or global vs. host culture identification (e.g., in migrants) have not been considered in tandem.

Finally, with some recent exceptions involving university (Hamer et al., 2021; Feng et al., 2022) and community (Buchan et al., 2011; Katzarska-Miller et al., 2012; Sparkman and Hamer, 2020; Chen et al., 2022) samples from diverse countries, and minority/underrepresented (Kunst and Sam, 2013; Koc and Vignoles, 2016; Bobowik et al., 2022) samples, general research exploring global identification has focused on WEIRD (Western, Educated, Industrialized, and Democratic) populations (see Henrich et al., 2010; Sparkman and Hamer, 2020). This creates generalization problems. Moreover, Tadmor et al. (2018) suggest that achieving the benefits of multicultural experiences may be difficult when these are perceived as costly or depleting. Immigrants develop new cultural identifications in contexts often characterized by prejudice, unequal power distribution and access to rights, and colonial laws. These difficulties may negatively impact the creative benefits that could derive from their acculturation processes. Literature needs to incorporate underrepresented and underprivileged samples, such as migrated individuals who often develop attachment to both a new host culture and to a global community (Arnett, 2002; Kunst and Sam, 2013), and explore the implications that their multiple cultural identifications may have on creativity.

### Present research

We examine the link between global culture identification and creativity, in two different samples residing in Barcelona (Spain): a host national (and largely student) sample and a diverse community sample of people of migrant origin. Given the existing mixed findings on the effects of global vs. ethnic national/local identity on creativity (Tidikis et al., 2018; Mehta, 2019), and the lack of research examining both cultural identifications comparatively, our goal was to study the contributions of both global and ethnic culture identifications on creativity. Moreover, drawing on previous literature on multicultural identities, we included the potential contribution of host culture identification to creativity in the immigrant sample. We expected global culture identification to be positively associated with creativity in both the immigrant (H1a) and the host national (H1b) samples. Additionally, we hypothesized that the process of acquiring a different cultural identity in the context of migration could also facilitate creativity, i.e., that host culture identification would be positively associated with creativity in the immigrant sample (H2). Finally, even though previous research on multicultural experiences points to a negative or null relationship between ethnic identification and creativity, some authors also suggest the opposite (i.e., a positive relationship). Thus, we took an exploratory approach to examine the association between ethnic identification and creativity, in both samples.

## Materials and methods

### Participants

The data derived from two different samples. The community immigrant sample consisted of 122 adults with immigrant background (59% females, *mean age* = 33 years, *sd* = 10.33, range 19 to 64). They resided in Barcelona, a bicultural and bilingual region of Spain in which two cultural identities coexist (Spanish and Catalan). Participants were born in Ecuador (*n* = 30, 66.7% females, *mean age* = 32, *sd* = 11.28), Morocco (*n* = 30, 63.3% females, *mean age* = 30, *sd* = 11.24), Pakistan (*n* = 31, 38.7% females, *mean age* = 29, *sd* = 8.31) or Romania (*n* = 31, 67.7% females, *mean age* = 38, *sd* = 8.32). A small group (*n* = 7) were born in Spain and had at least one parent born in one of the aforementioned countries. All participants had good working knowledge of one or both host languages (Catalan or Spanish), and had resided in Spain for a minimum of 5 years.

The host national sample consisted of 316 adults (55.7% females, *mean age* = 24 years, *sd* = 7.77, range 18 to 61), born in Spain. This was a predominantly student sample, since 75.3% (*n* = 238) of participants were studying at the time.

Additional sociodemographic characteristics for both the immigrant and the host national sample can be found in Supplementary Tables S1, S2.^5^

### Procedure

The data used in this article comes from two different studies, approved by the Ethics’ Committee of the University Pompeu Fabra. Data were collected at the University’s laboratory installations, *via* the Qualtrics platform. When *in situ* data collection was not possible, participants were offered to take the survey online.^6^ Participants were able to choose the language of preference (among the two host languages, i.e., Catalan or Spanish) to fill out the surveys. Details on the recruitment for both samples can be found in Supplementary material.

Data from the immigrant sample was collected in two stages. During the first stage (2012), a larger sample of participants (*N* = 216) was recruited *via* relevant migrant and cultural associations in the city of Barcelona, to participate in a social networks’ study that included some acculturation and identity variables (see Repke and Benet-Martínez, 2018). We aimed for a minimum of 200 participants to allow the possibility of detecting small to moderate effect sizes in our regression models. Sample size was determined by the possibilities of access to this particular population, as well as by time and money constraints. The second phase took place approximately 1 year later (2013–2014). Participants were re-contacted and invited to participate in a study that included measures of creativity, multicultural experiences, and intergroup attitudes. We were able to recruit 56.4% (*N* = 122) of the first stage study sample. Participants received monetary compensation (15 euros) for their participation in each study stage.

Data collection for the host national sample took place in 2015. The original study included an experimental manipulation designed to impact intergroup attitudes (that at the end yielded null effects). Besides assessing attitudes, it also measured creativity and included questions about multicultural experiences. Participants received monetary compensation (8 euros). Sample size was determined based on effect sizes from similar experimental studies.^7^

### Measures

#### Global, ethnic, and host culture identification

Drawing on previous studies (e.g., Benet-Martínez and Haritatos, 2005), we selected measures of self-identification with *global*, *ethnic,* and *host* (for immigrants) cultural communities. Participants were asked to indicate their degree of identification with each culture (“Please, indicate your degree of identification with the following cultures and communities, marking the answer that you consider more appropriate”), on a scale from 1 (*not at all*) to 7 (*very strongly*). Both samples indicated their degree of identification with a “global, international, world” culture. Ethnic culture identification referred to the country of origin (i.e., Ecuador, Morocco, Pakistan, or Rumania) for immigrants, whereas it was operationalized as identification with Catalan and Spanish cultures for host nationals. Immigrant participants were additionally asked about their identification with the host cultures (Catalan and Spanish cultures separately). Since all participants resided in a bicultural region of Spain, an average of the two local cultures (Catalan and Spanish) was computed, to create the indexes of ethnic (for host nationals) and host (for immigrants) culture identification.

#### Creativity

Both samples performed the Alternate Uses Task (Guilford, 1967), a widely used test that assesses divergent thinking and creativity (e.g., Leung and Chiu, 2008; Tadmor et al., 2012a). Participants were given 4 min to write as many uses as they could think of for common household items. The objects were a plastic bottle, a brick, and a cardboard box for the immigrant sample. The host national sample performed the task only with the plastic bottle. Following previous literature (Tadmor et al., 2012a,b), responses were coded in each study by a team of two raters, for (a) *fluency* (i.e., number of uses generated, per object), (b) *flexibility* (i.e., number of different use categories generated, per object), and (c) *originality* (i.e., originality mean of the different uses generated). Flexibility captured the breadth of categories represented in each object, generated *via* discussion between the coders (see Leung and Chiu, 2008; Tadmor et al., 2012a).^8^ Originality was operationalized as the combination of novelty and usefulness, following Amabile (1983), and it was evaluated on a scale from 1 (*not at all original*) to 5 (*extremely original*). Since the immigrant sample generated uses for three objects, we used the average scores of fluency, flexibility and originality across the three objects. Interrater reliabilities (intraclass correlation) were calculated for all dimensions. For the immigrant sample, a high interrater reliability coefficient was achieved for fluency (ICC = 0.83), flexibility (ICC = 0.91), and originality (ICC = 0.78) in a first subsample of 54 individuals. The remaining responses were coded by one of the two raters. For the host national sample, a high interrater reliability was achieved for fluency (ICC = 0.99), flexibility (ICC = 0.98) and originality (ICC = 0.99) for a subsample of 141 participants rated by two coders. An average of both coders was used to get the final scores. The remaining responses were coded by one of the two raters. For subsequent analyses, we use a computed average of the standardized scores of all three dimensions (fluency, flexibility, and originality mean), which represents overall creativity (α_immigrant sample_ = 0.66, α_national sample_ = 0.69).

#### Sociodemographic control variables: Gender, age, and education

There is some evidence that certain sociodemographic factors, such as gender (Abraham, 2016), age (Binnewies et al., 2008; Frosch, 2011; Rietzschel and Zacher, 2015; Aytug et al., 2018), and education (see Simonton, 2000) can be associated with creativity. Since both samples differed in terms of these relevant characteristics, we included these as control variables in supplementary analyses. Gender was conceived as a binary variable (1 = “Male,” 2 = “Female”). Age was measured as a continuous variable. Education represented—for both samples—the highest educational level achieved, on a scale from 1 (*No formal education or less than 5 years of schooling*) to 9 (*PhD degree*).^9^

### Analytical strategy

We used correlation and regression analyses to examine the link between identity and creativity. We ran two regression models, one for each sample. For the immigrant sample, ethnic, host, and global culture identifications were introduced as predictors of creativity. For host nationals, ethnic and global culture identifications were used as predictors. We additionally tested the same models including relevant sociodemographic control variables (see Supplementary material).

## Results

### Descriptive data and correlations

Table 1 (for immigrants) and Table 2 (for host nationals) show means and standard deviations of each variable used in the study. Correlation results in Table 1 reveal significant positive relationships between host culture identification and creativity (*p* = 0.028), as well as between global culture identification and host culture identification (*p* = 0.001), for the immigrant sample. There were no significant correlations between ethnic identification and creativity or global culture identification. Contrary to our expectations, global culture identification and creativity were not correlated. In contrast, for the host national sample, Table 2 shows a significant positive correlation between global culture identification and creativity (*p* = 0.026), and no other significant relationships.

**Table 1:** Means, standard deviations, and bivariate correlations between variables under study for the immigrant sample (*N* = 118/122).

	1	2	3	4
*M*	5.41	4.29	3.88	3.12
*SD*	1.58	1.21	1.76	1.05
1. Ethnic culture identification	1			
2. Host culture identification	0.07	1		
3. Global culture identification	0.09	0.31^**^	1	
4. Creativity	−0.04	0.20^*^	0.04	1

ID = Identification. Identification is measured on a 1 to 7 scale range. Creativity score is computed as an average of fluency, flexibility, and originality mean, across all three objects. We used pairwise deletion of missing values, thus the total sample size was between 118 and 122.

* *p* < 0.05;

** *p* < 0.01.

**Table 2:** Means, standard deviations, and bivariate correlations between variables under study for the host national sample (*N* = 312/315).

	1	2	3
*M*	4.56	4.49	5.36
*SD*	1.09	1.58	1.72
1. Ethnic culture identification	1		
2. Global culture identification	0.06	1	
3. Creativity	0.02	0.13^*^	1

ID = Identification. Identification is measured on a 1 to 7 scale range. Creativity score is computed as an average of fluency, flexibility, and originality mean. We used pairwise deletion of missing values, thus the total sample size was between 312 and 315.

* *p < 0.05*.

### Regression analyses

Results with global, ethnic, and host culture identifications predicting creativity are shown in Table 3 (for immigrants) and in Table 4 (for host nationals, with only global and ethnic culture identifications). Results confirm H1b, but not H1a. For the host national (but not the immigrant) sample, global culture identification significantly predicted creativity. For the immigrant sample, host culture identification significantly predicted creativity, supporting H2. There were no significant associations between ethnic culture identification and creativity, in any of the samples. Supplementary Table S3 (for immigrants) and Supplementary Table S4 (for host nationals) show results controlling for gender, age, and education level.^10^ Developing an identification that transcended the one acquired through enculturation (i.e., transcending Catalan or Spanish identification for host nationals, transcending ethnic identification for immigrants) was thus associated with creative behavior.

**Table 3:** Ethnic, host, and global culture identification predicting creativity among the immigrant sample (*N* = 118).

Variable	*B*	*SE*	β	*p*	*CI*_95%_
Ethnic culture identification	−0.01	0.05	−0.01	0.87	[−0.10, 0.08]
Host culture identification	0.12	0.06	**0.20**	0.04	[0.00, 0.24]
Global culture identification	−0.01	0.04	−0.02	0.83	[−0.09, 0.07]

ID, Identification. Statistically significant coefficients are shown in bold. The dependent variable (creativity) was computed as the average of the standardized scores of fluency, flexibility, and originality mean. B, unstandardized regression coefficient; SE, standard error; β, standardized regression coefficient; CI_95%_, Confidence interval. Due to missing values, the total sample size was *N* = 118.

**Table 4:** Ethnic and global culture identification predicting creativity among the host national sample (*N* = 311).

Variable	*B*	*SE*	β	*p*	*CI*_95%_
Ethnic culture identification	0.01	0.04	0.02	0.777	[−0.06, 0.08]
Global culture identification	0.06	0.03	**0.12**	0.028	[0.00, 0.11]

ID, Identification. Statistically significant coefficients are shown in bold. The dependent variable (creativity) was computed as the average of the standardized scores of fluency, flexibility, and originality mean. B, unstandardized regression coefficient; SE, standard error; β, standardized regression coefficient; CI_95%_, Confidence interval. Due to missing values, the total sample size was *N* = 312.

## Discussion

This research contributes to the scarce work investigating the link between creativity and identification with a global culture, in tandem with host and ethnic culture identification. We examined this link in two samples, including a diverse community immigrant sample—a type of population underrepresented in the literature on both global identification and creativity. Our findings partially support (for the host nationals) the hypothesis that a stronger global (vs. ethnic) culture identification would be associated with more creativity, in line with some previous research (Tidikis and Dunbar, 2019). However, our results also show that host (vs. global or ethnic) culture identification is positively linked to creativity among immigrants. Finally, we did not find any significant relationship between ethnic identification and creativity in any of the samples, in line with research on the positive impact of the acquisition of new and different cultural identities on creativity.

Our results suggest that developing an identity that transcends the one acquired through enculturation (i.e., going beyond one’s ethnic origin identity) has the potential of facilitating creative thinking processes. In line with the literature clarifying the role of multicultural experiences in creative performance (Leung et al., 2008; Ritter et al., 2012; Cheng and Tan, 2017; Dunne, 2017; Gocłowska et al., 2018; Bobowik et al., 2020, manuscript in preparation^11^), the development of higher levels of other-culture identification is associated with the ability to provide creative solutions to everyday life problems, because it results from constant contact with unfamiliar cultural information. This includes both direct contact (i.e., interactions) with a different culture (e.g., culture of settlement in the case of immigrants), and indirect contact (i.e., exposure) with any culture of the world through cultural products such as literature, movies, music, dance, or food (see Aytug et al., 2018; for a review, see Maddux et al., 2021). Moreover, both interactions with culturally diverse others and exposure to cultural diversity appear to contribute to identifying oneself as part of a global community (Sparkman and Hamer, 2020; Reysen, 2022).

Hence, we argue that for host nationals with limited abroad experiences, the predominant identification process derives from globalization-based acculturation (Chen et al., 2008; Kharkhurin, 2011) acquired through indirect exposure to different cultures. This results in global culture identification positively impacting creativity processes. In contrast, for immigrants, the predominant acculturation process involves attachment to the culture(s) of the new country of residence. Thus, host culture identification more significantly and directly impacts creativity. Recent research finds that a diverse social network predicts global culture identification in immigrants, and this relationship is mediated by a higher degree of integration of their ethnic and host culture identities (Bobowik et al., 2022). Globalization-based acculturation may thus in their case happen primarily through direct interactions (and identification) with culturally diverse others.

In this line, developing a host cultural identity (and integrating it with one’s ethnic identity) may be a first step to build a sense of global culture identity for migrant individuals. Our correlational results preliminarily support this assumption, by revealing a positive association between host and global culture identifications. Previous literature shows that *hybrid* or *balanced* identities (that embrace and integrate home and host cultures) are linked to global identity configurations (Sussman, 2000; Kohonen, 2008). Specifically, Kohonen (2008) equates a balanced identity pattern to an intercultural or global identity pattern of “world citizens” who are able to manage and interact in different cultural settings (see Sussman, 2000). Furthermore, previous research states that creativity increases when individuals integrate and feel personally attached to their different cultural identities (for a review, see Cheng and Tan, 2017). Future research should examine the association between ethnic, host and global culture identifications over time (e.g., longitudinal studies), and explore the contexts in which other-culture or balanced identity configurations precede (or not) global culture identification, and, in turn, creativity.

Finally, our results do not find any significant relationship between ethnic culture identification and creativity, in any of the samples. In other words, ethnic identification proves irrelevant for our samples’ creative skills, which again reinforces previous evidence on the potential of diversifying experiences (e.g., multicultural) in loosening category boundaries, attenuating the rigidity of the human mind, and ultimately boosting divergent thinking (Kharkhurin, 2011; Gocłowska et al., 2018). Our creativity task consisted of generating novel and unrelated ideas from diverse categories, and thus it rewarded divergent thinking, or the capacity to draw on different conceptual categories. Future studies should test this relationship using different creativity measures.

### Limitations and future directions

We acknowledge the limitations of our findings related to the immigrant sample, due to its non-random nature and its size. Moreover, it is a diverse sample in itself (i.e., participants from four different national origins), composed of people that had been in Spain for at least 5 years and had a good working knowledge of the host language.^12^ Thus, our results may not generalize to migrant populations with other characteristics. However, research on global identification or creativity with migrant populations is scarce, and we would like to emphasize the value of this sample. First, migrating comprises a deep multicultural experience that has the potential to strongly affect both individuals’ identity configurations and creativity, so one would expect scholars in these areas of research to incorporate migrant samples. Second, social psychology’s findings are overly informed by WEIRD populations. This undermines generalization, contributes to the replication crisis (Henrich et al., 2010) and offers a limited ethnocentric view of psychological phenomena. Third, people who migrate exercise creativity in their everyday lives by adapting to an unfamiliar culture while challenging the status quo and culturally dominant behavior patterns (Dixon et al., 2010; Bobowik et al., 2020, manuscript in preparation^13^). Consequently, they may bring new and diverse forms of creativity (e.g., problem-solving skills) to the settled societies (Franzoni et al., 2014; Shao et al., 2019). Future studies should provide more data on global identification and creativity with migrant populations.

Although the online recruitment system applied with host nationals allowed us to implement a simple random sampling methodology and to obtain a gender-balanced sample of people born in Spain, the pool was mainly composed by young university students. Even though we were interested in comparing this type of sample - typically found in social-psychological research - to a more diverse, underrepresented, and non-WEIRD type of sample, its student nature limits the generalizability of our results. Future studies should include large, representative and diverse samples in order to test and build on our current findings.

As an additional limitation, culture may have influenced the precise assessment of creativity. According to Shao et al. (2019), culture may influence: (a) the expression of creativity in individuals’ outputs (e.g., due to cultural familiarity with the creativity instrument), and (b) the subjective assessment of creativity (which is related to the cultural background of raters). In relation to the first point, the instrument used in our study (i.e., the Alternate Uses Task, by Guilford, 1967) was developed in the United States, and so it may have biased performance in favor of the host national (Western) sample. Moreover, language—as an integral aspect of culture—can influence the generation of creative output expressed in verbal forms (Shao et al., 2019). In this line, and even though immigrant participants had a good command of either one or the two host languages, they may have still been disadvantaged in comparison to the host national participants. With regards to the second point made by Shao et al. (2019), the team of creativity coders were from the dominant Western culture. Even though we tried to take an emic-etic approach (Berry, 1999) when coding creativity, by keeping in mind the universe of uses generated by the same cultural group (criterion adapted from Goclowska et al., 2012),^13^ coders’ cultural background may have biased results in favor of host nationals. Future studies should more carefully acknowledge and control for the influence of culture in the measurement of creativity, for example by using raters that match the cultural background of their study participants (for some examples, see Sun, 2018; and Tidikis et al., 2018).

Moreover, in this study we used a single item measure to assess each of the cultural identifications. Other researchers could further expand on these findings by using different and broader assessments of global identity—such as the IWAH scale; (McFarland et al., 2012), or by distinguishing how the two components of identification with humanity (i.e., bond and concern, see Hamer et al., 2021) differently contribute to each dimension of creativity.

Finally, we cannot determine causality from our research design. Even though our study does not measure creative personality, it may be easier for creative people to develop broader or other-culture identifications, or perhaps there is a bidirectional relationship. Our results including control variables suggest that, for the immigrant sample, education might be a more significant predictor of creativity than host culture identification. Although we controlled for some previously identified predictors of creativity (i.e., gender, age, education), other uncontrolled variables could influence creativity as well (e.g., level of acculturation stress, previous multicultural experiences, openness to experience). Future research could use experimental or longitudinal designs to help establish causal relations between identification measures and creativity.

## Conclusion

One of the outcomes of living in a highly interconnected and globalized world is the development of identifications that transcend our own cultural origin and that include culturally diverse others. Identification with a global culture is considered one of the more inclusive and superordinate levels of self-categorization (see McFarland et al., 2019). Cultural identifications become multiple and complex in the face of globalization and multicultural contexts. For example, migrants may preserve their ethnic culture while developing attachment to the host dominant culture and to a global culture (Arnett, 2002; Kunst and Sam, 2013). The present study contributes to the yet scarce research investigating the link between global culture identification and creativity, in comparison to national (ethnic and host) identifications. Importantly, it explores this relationship in a typical national sample and in a highly underrepresented and diverse community immigrant sample. Results differ depending on the sample; global culture identification is linked to creativity among host nationals, and host culture identification is associated with creativity among immigrants. For both samples, results suggest that creative thinking may result from the process of acculturation, in which new and different cultural identities are formed.

## Data availability statement

The raw data supporting the conclusions of this article will be made available by the authors, upon request.

## Ethics statement

The studies involving human participants were reviewed and approved by Parc de Salut MAR—Clinical Research Ethics Committee. The participants provided their written informed consent to participate in this study.

## Author contributions

ES conceptualized the paper, participated in the study design and data collection, analyzed the data, and drafted the article. MB conceptualized the paper, provided crucial feedback on the analytical strategy and the preliminary results, analyzed the data, edited the manuscript, and revised it critically, adding important intellectual content. VB conceptualized the paper, was responsible for the study design and data collection, provided feedback on the preliminary results, and provided critical edits and comments on the manuscript. All authors contributed to the article and approved the submitted version.

## Funding

This research was supported with funds received from RecerCaixa (2013–2015), with funds referenced PSI2016-79300-R from the “Ministerio de Economía y Competitividad” (MINECO), “Agencia Estatal de Investigación” (AEI), and the European Regional Development Fund (FEDER), and with funds received from the “Agència de Gestió d’Ajuts Universitaris i de Recerca” (AGAUR) of the Government of Catalonia, Spain (2010 ARAFI 100022).

## Conflict of interest

The authors declare that the research was conducted in the absence of any commercial or financial relationships that could be construed as a potential conflict of interest.

## Publisher’s note

All claims expressed in this article are solely those of the authors and do not necessarily represent those of their affiliated organizations, or those of the publisher, the editors and the reviewers. Any product that may be evaluated in this article, or claim that may be made by its manufacturer, is not guaranteed or endorsed by the publisher.

## Footnotes

^1^Still, being exposed to and learning from a new culture do not necessarily produce identification with that culture (Hong et al., 2007; Cheng et al., 2014). Individuals have a cultural identity when they express their attachment to that particular culture (Cheng et al., 2014).

^2^Compared to monocultural individuals, dual-identity individuals learn to alternate between their different identities (see cultural-frame switching; Benet-Martínez et al., 2002), reconciling discrepant values or cognition, and broadening their self-definition (Crisp and Gocłowska, 2014).

^3^The personality trait of openness to experiences has also been identified as a predictor of superordinate identifications such as IWAH (Hamer et al., 2019). For a review of both predictors and outcomes of global human identification, see McFarland et al. (2019).

^4^According to Mehta (2019) and Sun (2018), individuals who strongly identify with a national local culture are more faithful and respectful of local traditions, and strongly value relationships within their local community. This translates into a high relationship focus that makes them better at generating relationships between loosely connected ideas, which is also the basis for creativity.

^5^The immigrant sample has been used in previous studies (i.e., Repke and Benet-Martínez, 2018; Bobowik et al., 2021 2022).

^6^Only 13.1% of the immigrant participants and 13.8% of the host national participants chose the online method.

^7^Sample size for a 2 × 2 experimental design (ANOVA, fixed effects) was determined using the G.Power calculator (Faul et al., 2009). It was established that, in order to detect effect sizes similar to the ones found in relevant literature (between *d* = 0.36 and *d* = 0.8) with 80% power (alpha = 0.05, two-tailed), we required a minimum total sample of between 52 (*d* = 0.8) and 245 (*d* = 0.36) participants.

^8^Examples of categories for the plastic bottle were: liquid container, lighting, weapon, clothing and accessories, sound, and music.

^9^The educational level items were adapted from the International Standard Classification of Education, by the UNESCO Institute for Statistics (2012).

^10^For immigrants, the effect of host culture identification became marginally significant (*p* = 0.067) after controlling for gender, age, and education (see Supplementary Table S2). For host nationals, the effect of global culture identification remained significant after including controls (see Supplementary Table S4).

^11^Bobowik, M., Benet-Martínez, V., Repke, L., and Soler-Pastor, E. (2020). The role of intercultural and intracultural social networks for creativity among immigrants.

^12^We included these requirements to ensure that participants would clearly understand the survey questions and elaborate on the answers, and to minimize the potential "language advantage" of the host national sample in the creativity task. Moreover, 5 years was considered sufficient for developing identification to the host culture.

^13^For example, one use could be very common in Morocco but more novel and thus original in Ecuador.
