# Supplementary material for: Creativity and (global, ethnic, host) cultural identifications: An examination in migrant and host national samples
Source: Front Psychol. 2022 Nov 4;13:1007034. doi: 10.3389/fpsyg.2022.1007034 (PMC9672462; doi:10.3389/fpsyg.2022.1007034)
Supplement: Supplementary file 1 [file Data_Sheet_1.PDF]

Supplementary Material

1 Supplementary Data

1.1 Recruitment Details

Recruitment of immigrant participants during the first stage data collection (2012) was done via relevant migrant and cultural associations in the city of Barcelona, which facilitated access to this particular population (see Repke & Benet-Martínez, 2018). Thus, this was a non-probability sample (*N* = 216). During the second stage study (2013-2014), we recontacted all the sample. We took an extra effort to recruit as much participants as possible in a balanced subsample (across the different cultural groups), and to minimize nonparticipation via different strategies (e.g., economic incentives, diversifying the methods of contact). We were able to recruit 56.4% of the first stage sample (*N* = 122), which we believe is an acceptable percentage considering the difficulties of access to this type of sample.

The host national sample was recruited via the university's online recruitment system (www.orsee.org). This online system included a pool of over 5000 (mainly student) registered individuals and allowed to implement a simple random sampling methodology among this population, and to obtain a gender-balanced sample of people born in Spain.

2 Supplementary Tables

2.1 Table S1. Sociodemographic characteristics of immigrant sample. by ethnic origin (*N* = 122)

	Ecuador (*N* = 30)		Morocco (*N* = 30)		Pakistan (*N* = 31)		Romania (*N* = 31)		Total (*N* = 122)	
	n	%	*n*	%	*n*	%	*n*	%	*n*	%
Gender										
Male	10	33.3	11	36.7	19	61.3	10	32.3	50	41.0
Female	20	66.7	19	63.3	12	38.7	21	67.7	72	59.0
Age range										
19 to 34 years	16	53.3	20	66.7	22	71.0	9	29.0	67	54.9
35 to 49 years	12	40.0	9	30.0	8	25.8	21	67.7	50	41.0
50 to 64 years	2	6.7	1	3.3	1	3.2	1	3.2	5	4.1
Education										
None or Primary	2	6.7	3	10.0	3	9.7	1	3.2	9	7.4
Secondary	8	26.7	11	36.7	4	12.9	7	22.6	30	24.6
Vocational training or Incomplete university	18	60.0	11	36.7	12	38.7	17	54.8	58	47.5
University	2	6.7	5	16.7	12	38.7	6	19.4	25	20.5
Income										
< 1000 €	19	67.9	14	56.0	17	54.8	10	32.3	60	52.2
1001 – 2000 €	7	25.0	8	32.0	10	32.3	15	48.4	40	34.8
> 2000 €	2	7.1	3	12.0	4	12.9	6	19.4	15	13.0

Note. There were *N* = 7 participants (*n* = 2 from Ecuador. *n* = 5 from Morocco) that did not respond to the income question.

2.2. Table S2. Sociodemographic characteristics of the host national sample (*N* = 316)

	Total (N = 316)	
	n	%
Gender		
Male	140	44.3
Female	176	55.7
Age range		
18 to 33	278	88.3
34 to 48	25	7.9
48 to 61	12	3.8
Education		
Primary	1	0.3
Secondary	1	0.3
Vocational training or Incomplete university	218	69.0
University	96	30.4
Income		
< 1000 €	40	12.7
1001 – 2000 €	75	23.9
> 2001 €	199	63.4

Note. There were *N* = 1 missing response for age and *N* = 2 missing responses for income.

2.3 Table S3. Ethnic, host and global culture identification predicting creativity among the immigrant sample, controlling for gender, age and education (*N* = 117)

Variable	*B*	*SE*	*β*	*p*	$CI_{95\%}$
Ethnic culture identification	-.01	.05	-.02	.84	[-.10, .08]
Host culture identification	.11	.06	*.18*	.07	[-.01, .23]
Global culture identification	-.00	.04	-.01	.92	[-.09, .08]
Gender	.02	.14	.02	.87	[-.26, .31]
Age	.00	.01	.01	.90	[-.01, .01]
Education	.08	.04	*.18*	.05	[.00, .17]

Note. ID = Identification. Marginally significant coefficients are shown in italics. The dependent variable (creativity) was computed as the average of the standardized scores of fluency, flexibility, and originality mean. Gender was measured as a binary variable (1 = “Male”, 2 = “Female”). Age was measured as a continuous variable. Education was measured as a categorical variable, on a scale from 1 (*no formal education*) to 9 (*PhD degree*). *B* = unstandardized regression coefficient; *SE* =

standard error; β = standardized regression coefficient; $CI_{95\%}$ = Confidence interval. We used pairwise deletion of missing values, thus the total sample size was *N* = 117.

2.4 Table S4. Ethnic and global culture identification predicting creativity among the host national sample, controlling for gender, age and education (*N* = 310)

Variable	*B*	*SE*	*β*	*p*	$CI_{95\%}$
Ethnic culture identification	.01	.04	.01	.85	[-.07, .08]
Global culture identification	.06	.03	**.13**	.03	[.01, .11]
Gender	-.06	.09	-.04	.52	[-.23, .11]
Age	-.01	.01	-.06	.36	[-.02, .01]
Education	.05	.07	.05	.43	[-.08, .18]

Note. ID = Identification. Statistically significant coefficients are shown in bold. The dependent variable (creativity) was computed as the average of the standardized scores of fluency, flexibility, and originality mean. Gender was measured as a binary variable (1 = “Male”, 2 = “Female”). Age was measured as a continuous variable. Education was measured as a categorical variable, on a scale from 1 (*no formal education*) to 9 (*PhD degree*). *B* = unstandardized regression coefficient; *SE* = standard error; β = standardized regression coefficient; $CI_{95\%}$ = Confidence interval. We used pairwise deletion of missing values, thus the total sample size was *N* = 310.
